# Growth differentiation factor 15: an emerging diagnostic biomarker of liver fibrosis in chronic hepatitis C patients

**DOI:** 10.1186/s43066-021-00075-x

**Published:** 2021-01-19

**Authors:** Heba M. Adel Abou Zaghla, Aziza Ahmed El Sebai, Ossama Ashraf Ahmed, Ayat Fawzy Ahmed, Azza Abdel Rahman Saab

**Affiliations:** 1 Clinical Pathology, Ain Shams University, Cairo, Egypt; 2 Internal Medicine, Faculty of Medicine, Ain Shams University, Cairo, Egypt

**Keywords:** Liver fibrosis, Growth differentiation factor 15 (GDF-15)

## Abstract

**Background:**

Chronic liver disease and cirrhosis are of the major health concern worldwide. Assessment of liver fibrosis is necessary to determine disease severity and prognosis at the time of presentation to determine suitable treatment. Liver biopsy is considered as standard golden method in diagnosis of liver fibrosis. However, this procedure is invasive; thus, multiple laboratory and radiologic tests are used to help determination of the degree of fibrosis. Growth differentiation factor 15 (GDF-15) is a pleiotropic cytokine involved in regulating inflammatory and apoptotic pathways. It is suggested that GDF-15 plays an important role in pathogenesis of liver fibrosis. In this study, we aimed to evaluate efficiency of growth differentiation factor 15 in diagnosing liver fibrosis. The study was a case-control study conducted on 55 chronic HCV patients recruited from hepatitis C virus clinic at Faculty of Medicine Ain Shams Research Institute (MASRI), and 30 healthy subjects age- and sex-matched. The patients were classified into three subgroups according to the degree of liver fibrosis assessed by fibro-scan. Serum concentration of GDF-15 was determined by enzyme-linked immunosorbent assay.

**Results:**

Our results revealed a highly significant statistical rise in GDF-15 levels among studied chronic HCV patients with liver fibrosis when compared to the control group (*p* < 0.01). Furthermore, there was a significant positive correlation between the degree of fibrosis assessed by fibro-scan and GDF-15 serum levels. Levels of GDF-15 were significantly higher in patients with mild degree of fibrosis (patients’ subgroup І) when compared with the controls’ group (*p* < 0.01) suggesting the role of this marker in early detection of liver fibrosis. A statistically significant increase in serum GDF-15 levels was noticed among patients with advanced fibrosis “subgroup ІІІ” compared to those with mild fibrosis “subgroup І” (*p* < 0.05). The diagnostic sensitivity and specificity of GDF-15 were 96.7%, 98.2%, respectively at a cut-off value of 150 ng/L for discrimination between patients’ and controls’ groups.

**Conclusion:**

Growth differentiation factor 15 could be a potential marker of liver fibrosis especially in early detection as its levels were significantly higher in patients’ group with liver fibrosis than controls’ group and there was a significant positive correlation between the degree of liver fibrosis and GDF-15 serum levels.

## Background

Liver fibrosis is a common pathological consequence of a variety of chronic stimuli including viral, autoimmune, drug induced, cholestatic, and metabolic diseases. Advanced liver fibrosis results in cirrhosis, portal hypertension, hepatocellular carcinoma, and liver cell failure [1].

Chronic liver disease has high global mortality rates. Egypt has a high hepatitis C virus (HCV) prevalence, where 11.5% of estimated populations are HCV positive [2]. Assessment of liver fibrosis helps clinicians to predict patient prognosis, to initiate treatment at early stage of fibrosis and to achieve high survival rate [3].

The degree of liver fibrosis is determined mainly by liver biopsy, but issues regarding its invasiveness and the small amount of liver tissue evaluated limit its applicability [4]. On the other hand, transient elastography (fibro-scan) is one of the most successful methods for assessment of liver fibrosis due to its noninvasive nature, reproducibility, and high diagnostic performance providing a quantifiable estimate of liver stiffness in unit known as kilopascals. Fibroscan measures liver stiffness in a volume of approximately a cylinder of 1-cm diameter and 5-cm long, which is roughly 100 times the volume of a percutaneous liver biopsy*.* In HCV fibroscan results ≤ 7 kPa account for F0–F1, results ≥ 7.1–8.6 kPa account for F1–F2, results ≥ 8.7–9.4 kPa account for F2, results ≥ 9.5–12.4 kPa accounts for F3, results ≥ 12.5–14.4 kPa account for F3–F4, results ≥ 14.5 kPa accounts for cirrhosis [5]. However, fibro-scan has some limitations in individuals with narrow intercostal spaces, morbid obesity, and increased liver stiffness for causes rather than fibrosis [6].

A series of serum markers of liver fibrosis had been developed. They are classified into direct and indirect markers. Direct markers refer to molecules involved in hepatic fibrogenesis and extracellular matrix turnover such as hyaluronic acid and Procollagen N-terminal peptide, while indirect markers involve molecules that reflect liver function such as ALT, AST, and GGT. Direct and indirect markers can be used alone or combined to form composite scores, e.g., AST-platelet ratio index (APRI), fibrosis-4 index (FIB-4 index), and hepascore. However, most of these markers are unable of accurate distinction of fibrosis stage especially early stages [7].

In the past few years, growth differentiation factor 15 (GDF-15) was the matter of research by many scientists. It is a transforming growth factor β (TGF-β) protein related to infection, fibrosis, and apoptosis pathways in case of tissue damage or disease. Its mRNA is known to be found particularly in the liver. In the presence of hypoxia, anoxia, inflammation, radiation exposure, and tissue injuries, the GDF-15 gene is expressed by activated macrophages which increase synthesis of GDF-15 protein [8]. Immediate induction of GDF-15 is an initial response to liver injury which can happen via TNF and p53 dependent and independent processes. Direct liver injury can induce GDF-15 expression in hepatocytes in the absence of other cell types such as inflammatory cells. GDF-15 has been put forward as a predictive biomarker of liver fibrosis and severity in patients with chronic liver disease. As a result of chronic damage to hepatocytes, prolonged stimulation of hepatic stellate cells results in the release of profibrogenic abundant factors such as GDF-15, leading to the development of liver cirrhosis. GDF-15 was found to not only stimulate transforming growth factor beta 1 (TGF-β1) expression, but it also induces fibrosis by directly increasing phosphorylation of SMAD2 and SMAD3, which play a crucial role in HSC activation and fibrogenesis. GDF-15 leads to increase expression of fibrosis markers, such as α-SMA and collagen I. Also, it leads to ECM accumulation by inhibition of tissue collagenases expression as well as increasing synthesis of tissue inhibitors of metalloproteinases [9].

## Aim of the work

The aim of this work was to investigate the clinical utility of GDF-15 serum level as a predictor of the degree of liver fibrosis in patients with chronic HCV infection and correlation of its serum level with the fibro-scan value.

## Methods

This case-control study was conducted from January 2019 to February 2020 on 55 HCV-positive patients who underwent fibro-scan for assessment of liver fibrosis. They were recruited from hepatitis C virus Clinic at Faculty of Medicine Ain Shams Research Institute (MARSI). In addition, 30 age- and sex- matched apparently healthy subjects served as healthy controls. The study was in accordance with the Declaration of Helsinki (2013). All participants gave their written consent to enter the study. The study has been approved by the ethical committee of Faculty of Medicine, Ain Shams University.

### Subjects were classified into the following groups

#### Patient group (*n* = 55)

This group included 55 patients undergoing fibro-scan for assessment of liver fibrosis caused by chronic HCV infection. They were 35 males and 20 females. Their ages ranged from 27 to 75 years. They were further divided into the following subgroups according to the degree of fibrosis.

##### Subgroup І “F0 and F1” (*n* = 18)

This group included 18 patients with fibrosis stage of F0 and F1 with liver stiffness measure (LSM) less than or equal 7 kPa. Their ages range from 26 to 67 years.

##### Subgroup II “F2” (*n* = 13)

This group included 13 patients with fibrosis stage of F2 with LSM (7.1–9.4 kPa). Their ages range from 28 to 74 years

##### Subgroup III “F3–F4” (*n* = 24)

This group included 24 patients with fibrosis stag of F3 with LSM (9.5–12.4 kPa) and F4 with LSM (≥ 12.5 kPa). Their ages range from 38 to 75 years.

#### Control group (*n* = 30)

This group included thirty age- and sex-matched subjects. Their HCV antibody test was negative, and their pelvi-abdominal U/S showed no abnormality. They were 19 males and 11 females. Their ages range from 25 to 70 years.

Subjects with any of the following conditions were excluded from the study; HCV, alcohol abuse, evidence of autoimmune liver disease, BMI of more than 35 kg/m^2^, hepatocellular carcinoma or any extrahepatic malignancy and intake of any medication known to have injurious effect on the liver.

### All individuals in this study were subjected to the following

- Full history taking: focusing on chronic liver disease and its complications, smoking, alcohol, drugs, and any immunological disease.
- Thorough clinical examination with special emphasis on abdominal examination, presence of jaundice, edema, or ascites.
- Radiological investigations including transient elastography (for patients only) and pelvi-abdominal ultrasound.
- Laboratory investigations including:
  - Routine laboratory investigations; including complete blood count, fasting blood sugar, liver profile (total and conjugated bilirubin, serum ALT, AST, INR, total protein, and albumin), and alfa fetoprotein.
  - HCV Ab testing and polymerase chain reaction for HCV.
  - Assay of serum concentration of GDF-15 by enzyme-linked immunosorbent assay (ELISA).

### Analytical method of GDF-15

Growth differentiation factor-15 concentration was measured using a commercially available ELISA kit supplied by Shanghai Korain Biotech Co.., Ltd. The level of GDF-15 in samples was determined using a double-antibody sandwich ELISA. In this technique, GDF-15 is captured between two antibodies. The first is GDF-15 monoclonal antibody that was fixed to the inner wall of ELISA wells plate and the second is labeled with biotin to which Streptavidin-horse radish peroxidase (HRP) is combined forming immune complex. Addition of substrate results in color development that is stopped by acidic stopping solution. Absorbance of the developed color is measured spectrophotometrically at a wavelength of 450 nm. The concentration of GDF-15 is proportional to the intensity of the color of test sample. A standard curve is constructed from which the concentrations of GDF-15 in the samples are determined.

## Results

### Statistical analysis

IBM SPSS statistics (V. 26.0, IBM Corp., USA, 2019) was used for data analysis. *p* value > 0.05 will be considered statistically significant.

**Fig. 1:**
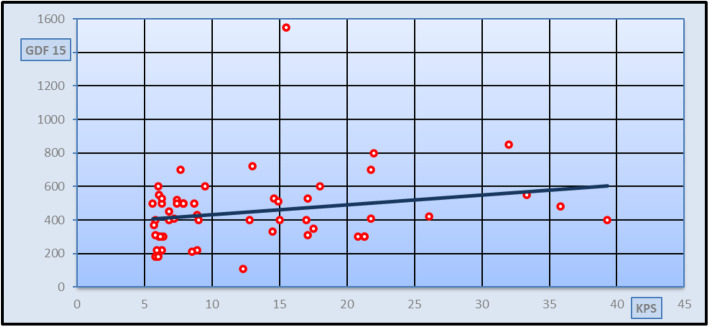
Correlation study between GDF-15 and fibroscan in KPS in all patients group [*r* = 0.086 and *p* = 0.034 (significant)]

**Fig. 2:**
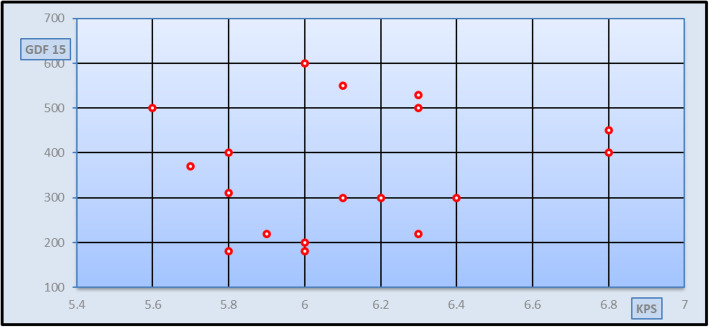
Correlation study between GDF-15 and fibroscan in KPS in group I patients [*r* = 0.15 and *p* = 0.553 (non-significant)]

**Fig. 3:**
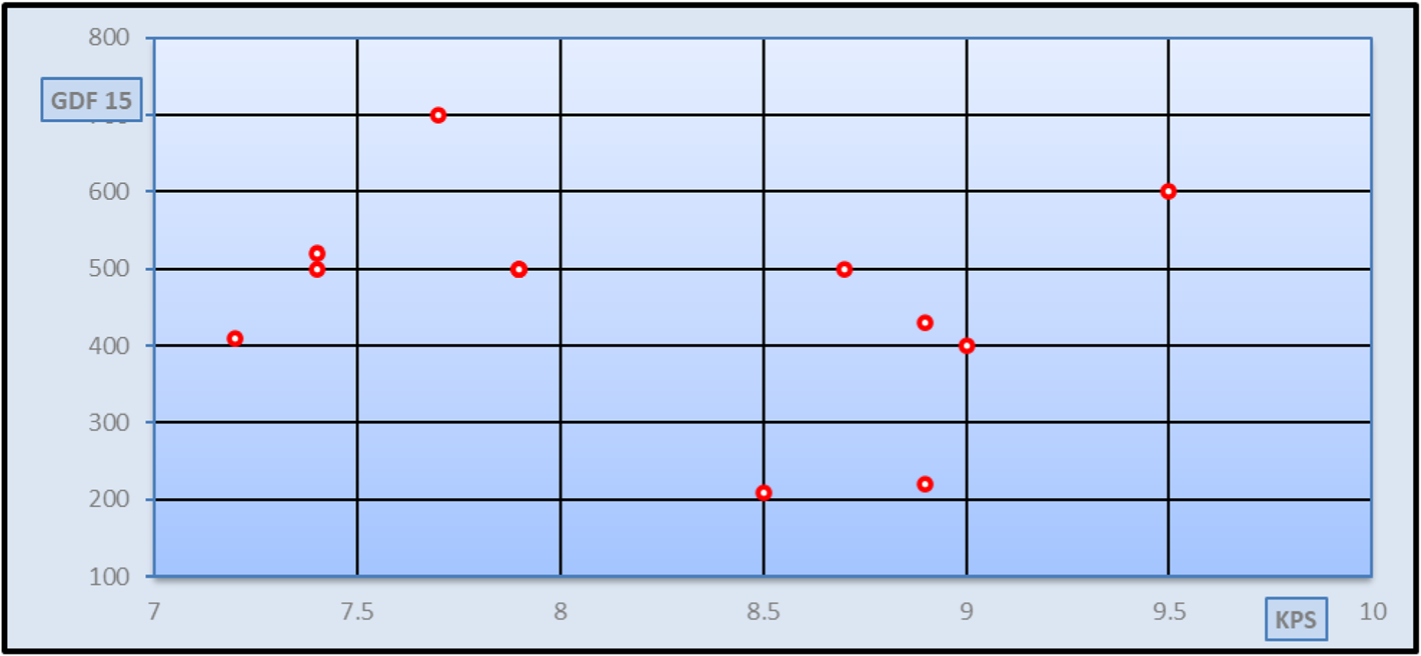
Correlation study between GDF-15 and fibroscan in KPS in group II patients [*r* = − 0.251 and *p* = 0.409 (non-significant)]

**Fig. 4:**
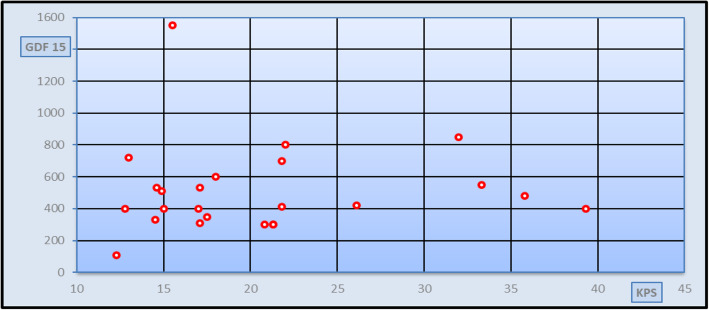
Correlation study between GDF-15 and fibroscan in KPS in group III patients [*r* = 0.203 and *p* = 0.342 (non-significant)]

**Fig. 5:**
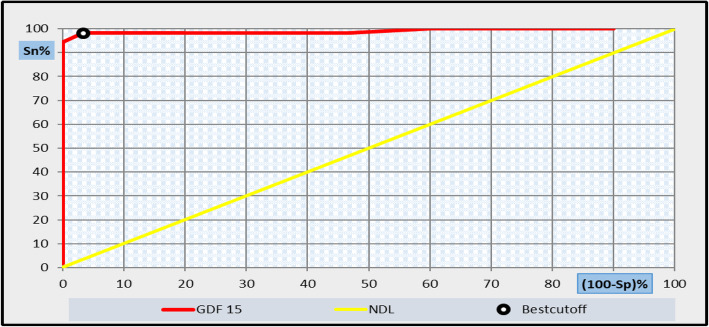
Receiver operating characteristic curve (ROC) analysis showing the diagnostic performance of GDF-15 for discriminating HCV patients with liver fibrosis from those control with area under the curve (AUC) = 0.99

**Fig. 6:**
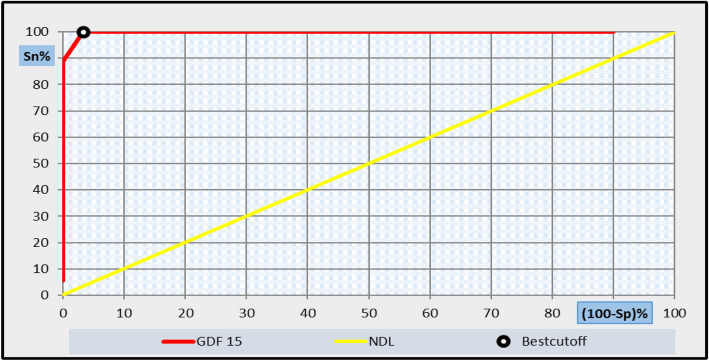
Receiver operating characteristic curve (ROC) analysis showing the diagnostic performance of GDF-15 for discriminating patients with subgroup-1 (F0–1) from those control with area under the curve (AUC) = 0.798

**Fig. 7:**
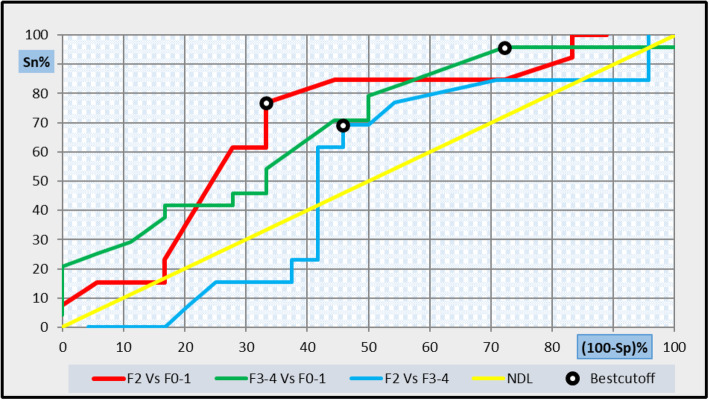
ROC curve analysis showing the diagnostic performance of GDF-15 for discriminating patients of different patients’groups

The results obtained in the present study are shown in Tables 1, 2, 3, 4, and 5 and Figs. 1, 2, 3, 4, 5, 6, and 7.

**Table 1:** Descriptive statistics of the different studied parameters in the control group

Parameter	Median (*n* = 30)	Q1–Q3 (*n* = 30)
Age (years)	31	27–46.25
Weight (kg)	76	69–87.25
AST (IU/L)	18	8–20
ALT (IU/L)	17	7–19
T. bilirubin (mg/dl)	0.7	0.5–0.8
D. bilirubin (mg/dl)	0.2	0.1–0.2
Albumin (g/L)	4.3	4–4.5
Platelets (10^3/ul)	240	190.25–260
INR	1	0.987–1.025
AFP (IU/ml)	2	1–4.25
GDF-15 (ng/L)	110	100–120

**Table 2:** Descriptive and comparative statistics of the different studied parameters in the different patient subgroups

Parameter	Liver fibrosis patient group (***n*** = 55)									Kruskal-Wallis test	
	Subgroup І F0-F1			Subgroup II F2			Subgroup III F3–F4			H	***p*** value
	***n***.	Median	Q1–Q3	***n***.	Median	Q1–Q3	***n***.	Median	Q1–Q3		
Age (years)	16	32	28–47.25	12	37.5	32–47.5	22	52.5	43.75–57.5	12.522	0.002
Weight (kg)	18	73.5	68–78.25	13	75	67.5–86.5	24	70	66.5–84.5	0.273	0.873
AST (IU/L)	18	28	21–36.25	13	25	19–43	24	50.5	8.75–72.5	20.873	0
ALT (IU/L)	18	27	20.25–43.5	13	35	21.5–48	24	47	35.5–64.25	9.367	0.009
T. bilirubin (mg/dl)	18	0.65	0.475–0.8	13	0.6	0.35–0.8	24	1	0.8–1.75	16.122	0
D. bilirubin (mg/dl)	18	0.2	0.1–0.2	13	0.2	0.1–0.3	24	0.3	0.2–0.7	13.276	0.001
Albumin (g/L)	17	4.1	3.95–4.45	13	4.2	4–4.5	24	3.55	3.2–4.1	14.857	0.001
Platelets (10^3/ul)	18	232	188.25–256	13	256	219–295.5	24	135	91–223.75	15.835	0
INR	18	1	0.987–1.025	13	1	1–1.1	23	1.1	1–1.3	10.895	0.004
AFP (IU/ml)	9	2.5	1.2–5.45	11	2.9	1.9–4.5	23	9.2	2.9–13.6	9.89	0.007
GDF-15 (ng/L)	18	340	220–500	13	500	405–510	24	415	335–587.5	5.185	0.075

Q1–Q3* = interquartile range (25th–75th percentiles); *p* value > 0.05 is non-significant; *p* value < 0.01 is highly significant

**Table 3:** Comparative statistics of GDF-15 between control group and whole patients’ group and between each two patients’ sub-groups using Wilcoxon’s rank sum test

**Group**	**Control (*n*** **= 30)**		**Patient group (*n*** **= 55)**		**Wilcoxon rank sum test**	
**Parameter**	**Median**	**Q1–Q3***٭*	**Median**	**Q1–Q3***٭*	***Z***	***p*** **value**
GDF-15 (ng/L)	110	100–120	410	300–530	− 7.442	0
**Group**	**Control (*n*** **= 30)**		**Patient subgroup I (*n*** **= 18)**		**Wilcoxon rank sum test**	
**Parameter**	**Median**	**Q1–Q3***٭*	**Median**	**Q1–Q3***٭*	***Z***	***p*** **value**
GDF-15 (ng/L)	110	100–120	340	220–500	− 5.753	0
**Group**	**Subgroup І F0–F1 (*n*** **= 18)**		**Subgroup II F2 (*n*** **= 13)**		**Wilcoxon rank sum test**	
**Parameter**	**Median**	**Q1–Q3***٭*	**Median**	**Q1–Q3***٭*	***Z***	***p*** **value**
GDF-15 (ng/L)	340	220–500	500	405–510	− 1.834	0.067
**Group**	**Subgroup І** **F0–F1 (*n*** **= 18)**		**Subgroup III** **F3–F4 (*n*** **= 24)**		**Wilcoxon rank sum test**	
**Parameter**	**Median**	**Q1–Q3***٭*	**Median**	**Q1–Q3***٭*	***Z***	***p*** **value**
GDF-15 (ng/L)	340	220–500	415	335–587.5	− 2.065	0.039
**Group**	**Subgroup II** **F2 (*n*** **= 13)**		**Subgroup III** **F3–F4 (*n*** **= 24)**		**Wilcoxon rank sum test**	
**Parameter**	**Median**	**Q1–Q3***٭*	**Median**	**Q1–Q3***٭*	***Z***	***p*** **value**
GDF-15 (ng/L)	500	405–510	415	335–587.5	− 0.08	0.936

Q1–Q3* = Interquartile range (25th–75th percentiles) *p* value > 0.05 is non-significant, *p* value < 0.01 is highly significant

**Table 4:** Three models of logistic multi regression analysis used to predict the most sensitive predictors of liver fibrosis

Module	Module 1				Module 2				Module 3			
Item	Reg. Coef.	***t***	***p***	Sig.	Reg. Coef.	***t***	***p***	Sig.	Reg. Coef.	***t***	***p***	Sig.
Platelets (10^3/ul)	− 0.003	− 1.605	0.126	NS	− 0.003	− 2.55	0.01	S	− 0.002	− 1.66	0.014	S
GDF-15 (ng/L)	0	− 0.463	0.649	NS	0.001	2.112	0.04	S	0.001	2.002	0.05	S
Weight (kg)	− 0.023	− 2.201	0.041	S	0.006	0.828	0.41	NS				
Prothrombin time (s)	0.21	1.609	0.125	NS	0.065	0.705	0.48	NS				
INR	− 2.712	− 1.917	0.071	NS	0.474	0.43	0.67	NS				
Age (years)	− 0.002	− 0.204	0.841	NS								
Height (cm)	0.016	0.885	0.388	NS								
AST (IU/L)	0.006	0.862	0.4	NS								
ALT (IU/L)	0.003	0.468	0.645	NS								
Bilirubin (mg/dl)	0.2	0.041	0.968	NS								
Direct bilirubin (mg/dl)	0.238	0.049	0.962	NS								
Indirect bilirubin (mg/dl)	0.431	0.089	0.93	NS								
Albumin (g/L)	− 0.017	− 0.065	0.949	NS								
Creatinine (mg/dl)	0.01	0.101	0.921	NS								
Fasting blood sugar (mg/dl)	− 0.003	− 0.818	0.424	NS								
Alfa fetoprotein (IU/ml)	0.016	0.803	0.433	NS								
*F* ratio	2.16				2.984				6.003			
*p*	0.059				0.02				0.005			
Sig.	Not significant				Significant				Highly significant			

*p* > 0.05 is non-significant; *p* < 0.05: significant; *p* < 0.001 highly significant

**Table 5:** Diagnostic performance of GDF-15 as a marker of liver fibrosis for discriminating different groups

GDF-15(ng/L) for discriminating HCV patients’ group from the control group	**Cut-off**	**Sensitivity (%)**	**Specificity (%)**	**NPV (%)**	**PPV (%)**	**EFF (%)**	**AUC**
	**150**	**96.7**	**98.2**	**96.7**	**98.2**	**97.6**	**0.99**
GDF-15(ng/L) for discriminating subgroup I patients from control group	**Cut-off**	**Sensitivity (%)**	**Specificity (%)**	**NPV (%)**	**PPV (%)**	**EFF (%)**	**AUC**
	**150**	**100**	**96.7**	**100**	**94.7**	**97.9**	**0.798**
GDF-15(ng/L) for discriminating subgroup І (F0–F1) patients from subgroup II (F2)	**Cut-off**	**Sensitivity (%)**	**Specificity (%)**	**NPV (%)**	**PPV (%)**	**EFF (%)**	**AUC**
	**400**	**66.7**	**76.9**	**80.8**	**62.5**	**71.0**	**0.694**
GDF-15(ng/L) for discriminating subgroup І (F0–F1) patients from subgroup III (F3–4)	**Cut-off**	**Sensitivity (%)**	**Specificity (%)**	**NPV (%)**	**PPV (%)**	**EFF (%)**	**AUC**
	**220**	**27.8**	**95.8**	**83.3**	**63.9**	**66.7**	**0.687**
GDF-15(ng/L) for discriminating subgroup II (F2) patients from subgroup III (F3–F4).	**Cut-off**	**Sensitivity (%)**	**Specificity (%)**	**NPV (%)**	**PPV (%)**	**EFF (%)**	**AUC**
	**420**	**54.2**	**69.2**	**76.5**	**45.0**	**59.5**	**0.508**

*AUC* area under the curve, *NPV* negative predictive value, *PPV* positive predictive value, *EFF* efficiency

Table 1 demonstrates the descriptive statistics of the different studied parameters in the control group.

Table 2 demonstrates the descriptive and comparative statistics of the various studied parameters between the three patients’ subgroups included in this study using Kruskal-Wallis test. Significant difference was revealed between the three subgroups regarding different studied parameters (age, AST, ALT, total and direct bilirubin, albumin, platelets count, INR, and AFP) (*p* < 0.05). Nine results of AFP were only available with subgroup I patients among 18 patients in this retrospective study (as they may be considered as low-risk patients for cancer liver). Borderline significant difference was revealed as regards GDF-15 (*p* value 0.075) among the three patients’ subgroups using Kruskal-Wallis test but by doing post hook test using Wilcoxon rank sum test between each two groups, levels of GDF-15 were significantly higher in subgroup III patients when compared with subgroup I patients. No significant difference was revealed among the three patients’ subgroups using Kruskal-Wallis test as regards weight (*p* value 0.8).

The statistical comparison of different studied parameters was done between each two patients’ subgroup using Wilcoxon rank sum test. A high significant difference was revealed between subgroups Ц and III patients, and between subgroups І and III patients regarding different studied parameters (age, AST, ALT, total and direct bilirubin, albumin, platelets count, INR, and AFP), *p* value < 0.01. While no significant difference was revealed between subgroups І and Ц patients regarding all studied parameters (*p* > 0.05). Also, no significant difference was revealed between each two patients’ subgroup regarding weight as *p* value > 0.05.

Table 3 demonstrates comparative statistics of GDF-15 level between different groups using Wilcoxon rank sum test. Levels of GDF-15 were significantly higher in all patients’ group when compared with the controls’ group (*p* value = 0). Also, levels of GDF-15 were significantly higher in subgroup I patients when compared with the controls’ group (*p* value = 0). Levels of GDF-15 were higher but not significantly in subgroup II patients when compared with subgroup I patients, and levels of GDF-15 were significantly higher in subgroup III patients when compared with subgroup I patients. Levels of GDF-15 were higher but not significantly in subgroup III patients when compared with subgroup II patients.

Spearman’s rank correlation analysis was done between different stages of fibrosis classified according to fibro-scan and measured in kilopascal (kPa) and the studied parameters in patients’ group and also, Spearman’s rank correlation analysis was done between GDF-15 serum levels and the studied parameters in patients’ group. As seen in Fig 1, a significant positive correlation was found between the degree of fibrosis and GDF-15 serum levels in all patients’ group (*r* = 0.286 and *p* value = 0.034), but no significant correlation was found between the degree of fibrosis and GDF-15 serum levels in different patients’ subgroups (Figs. 2, 3, and 4). Spearman’s rank correlation analysis also revealed a highly significant positive correlation between fibrosis degree and age, AST, ALT, and INR with *p* value < 0.01 and a highly significant negative correlation between fibrosis degree and both albumin and platelets count *p* value < 0.01. It was found a significant negative correlation between GDF-15 serum levels and the weight (*r* = − 0.314 and *p* value = 0.019).

Logistic regression was used as shown in Table 4 to estimate the association between the degree of liver fibrosis and different independent variables. It showed that the most sensitive independent variables to predict liver fibrosis were GDF-15 (*p* value 0.05) and platelets (*p* value 0.01).

Receiver operating characteristic (ROC) curve analysis was also applied to assess the diagnostic performance GDF-15 (ng/L) in diagnosis of liver fibrosis. The best-balanced cut-off level of GDF-15 that discriminates between chronic HCV patient with liver fibrosis and controls was 150 ng/L, at which sensitivity was 96.7%, specificity 98.2%, positive predictive value (PPV) 98.2%, negative predictive value (NPV) 96.7%, and the diagnostic efficiency was 97.6%, with an area under the curve (AUC) of 0.99 as shown in Fig. 5 and Table 5.

Receiver operating characteristic (ROC) curve analysis was applied to assess the diagnostic performance GDF-15 (ng/L) in diagnosis of liver fibrosis. The best-balanced cut-off level of GDF-15 that discriminates between subgroup І patients and controls was 150 ng/L, at which sensitivity was 100%, specificity 96.7%, positive predictive value (PPV) 94.7%, negative predictive value (NPV) 100%, and the diagnostic efficiency was 97.9%, with an area under the curve 0.798 as shown in Fig. 6 and Table 5.

Another ROC curve analysis was applied to assess the diagnostic performance GDF-15 for discriminating between subgroups І and Ц patients. The best diagnostic cut-off for GDF-15 was 400 ng/L, which had a diagnostic specificity of 76.9%, sensitivity 66%, NPV 80%, PPV 62.5%, and efficacy 71%. AUC was 0.694 as shown in Fig. 7 and Table 5. The combined use of GDF-15 and platelets count at cut-off 400 ng/L and 320,000/ul, respectively, achieved an increase in sensitivity (100%), specificity (84.6%), NPV 90%, PPV 100%, and efficacy 93.5% by multi-ROC analysis with AUC = 0. 897.

Also, another ROC curve analysis was applied to assess the diagnostic performance GDF-15 for discriminating between subgroups І and III patients. The best diagnostic cut-off for GDF-15 was 220 ng/L, with a diagnostic specificity of 95.8%, sensitivity 27.8%, NPV 83.3%, PPV 63.9%, efficacy 66.7%, and AUC 0.687 as shown in Fig. 7 and Table 5. An increase in sensitivity (100%), specificity (95.8%), NPV 94.7%, PPV 100%, and efficacy 97.6% was achieved after adding PLTs at cut-off of 330,000/ul by the multi-ROC analysis with AUC = 0.93.

The last ROC curve analysis was applied to assess the diagnostic performance of GDF-15 for discriminating between subgroups Ц and III patients. The best diagnostic cut-off for GDF-15 was 420 ng/L, with a diagnostic specificity of 69.2%, sensitivity 54.2%, NPV 76.5%, PPV 45%, efficacy 59.5%, and AUC 0.508 as shown in Fig. 7 and Table 5. Also, an increase in sensitivity (100%), specificity (92.3%), NPV (96%), PPV (100%), and efficacy (97.3%) was observed after adding PLTs at cut-off 254,000/ul by Multi-ROC analysis with AUC = 0.914.

## Discussion

Growth differentiation factor 15 (GDF-15), a distant member of the transforming growth factor β (TGF-β) superfamily, has been identified as a pleiotropic protein that plays key roles in fetal development, inflammation, regulation of cellular responses to stress signals, and in tissue repair [10].

Serum GDF-15 levels were significantly increased in patients with liver cirrhosis and hepatocellular carcinoma [11]. Furthermore, high serum GDF-15 level is associated with a risk of advanced fibrosis among NAFLD subjects [12].

Growth differentiation factor 15 leads to the extracellular matrix (ECM) accumulation directly by increasing the synthesis of ECM components as procollagen 1a and indirectly by inhibition of tissue collagenases expression and increasing synthesis of ECM-degrading enzyme inhibitors (as plasminogen activator inhibitor type 1 and tissue inhibitors of metalloproteinases) [8]. Thus GDF-15 has been put forward as a predictive biomarker of liver fibrosis and severity in patients with chronic liver disease [13].

The aim of this work was to study the clinical utility of GDF-15 serum level in prediction of the degree of liver fibrosis in patients with chronic HCV infection through correlation of its levels with fibrosis degree assessed by fibro-scan.

Results of our study revealed a highly significant statistical rise in GDF-15 levels among studied chronic HCV patients when compared to the control group. Similar results were obtained by Cheng et al. [11] who measured serum GDF-15 levels in 54 patients with chronic HCV. They reported that GDF-15 was associated with the pathogenesis of hepatitis C virus as a host response to viral proteins, infection-induced cell stress or both. Also, Abdulla et al. [14] reported that GDF-15 levels were increased in the serum of patients with cirrhosis and/or hepatocellular carcinoma compared with controls.

Subgroup І patients with mild degree of liver fibrosis showed significantly higher GDF-15 levels than control group (*p* value = 0), suggesting the role of this marker in early detection of liver fibrosis. A statistically significant increase in serum GDF-15 levels was noticed among patients with advanced fibrosis (subgroup ІІІ) compared to those with mild fibrosis (subgroup І), while there was no significant difference in the GDF-15 level among patient subgroups І and Ц and between patient subgroups Ц and ІІІ. Krawczyk et al. [15] assessed degree of liver fibrosis in 229 patients by both liver biopsy and fibro-scan and in 605 patients by fibro-scan only. They reported that GDF-15 could not discriminate between significant fibrosis (≥F2) and cirrhosis (≥F4) in patients staged with fibro-scan only.

The present study revealed a significant positive correlation between the degree of fibrosis assessed by transient elastography and GDF-15 serum levels in all patients’ group, but no significant correlation was found between transient elastography and GDF-15 in the three patients’ groups separately. These results are in contrast with those of Krawczyk et al. [15] who proved that there is a highly significant correlation between GDF-15 level and histological stages of fibrosis; this might be due to small sample size in each patients’ subgroup in our study. In addition, Kim et al. [8] found that patients with more severe chronic liver diseases had proportionately higher GDF-15 values. Furthermore, they stated that the increase in GDF-15 is caused by fibrosis rather than the hepatocellular damage by the infections in chronic liver diseases.

Also, our study showed a highly significant positive correlation between the degree of fibrosis and AST, ALT, total bilirubin, direct bilirubin and prothrombin time and a highly significant negative correlation between the degree of fibrosis and both albumin and PLTs count. Our study showed a significant negative correlation between GDF-15 levels and the weight. However, there was no significant correlation between GDF-15 and the other studied parameters.

In our study, a cut-off of 150 ng/L was found to discriminate both liver fibrosis degree of F0–1 from control and chronic HCV patients with liver fibrosis from controls. However, Kim et al. [8] who studied 246 Asian subjects, reported a cut-off of 574 ng/L for discriminating chronic hepatitis patients from controls. This discrepancy may be attributed to different performance of the used kits, our relatively small sample size, and difference between ethnic groups.

Also, ROC curve analysis was applied to assess the diagnostic performance of GDF-15 in detecting early fibrosis, and also discriminating between early and advanced fibrosis. The best diagnostic cut-off early detection of fibrosis for GDF-15 was 150 ng/L, which had a diagnostic specificity of 96.7%, sensitivity 100%, NPV 100%, PPV 94.7%, and efficacy 97.9%. The best diagnostic cut-off for discriminating between early and advanced fibrosis for GDF-15 was 220 ng/L, which had a diagnostic specificity of 95.8%, sensitivity 27.8%, NPV 83.3%, PPV 63.9%, and efficacy 66.7%. AUC was 0.687. Fortunately, the combined use of GDF-15 and platelets count at cut-off 220 ng/L and 330,000/ul respectively achieved in an increase in sensitivity (100%), specificity (95.8%), NPV (94.7%), PPV (100%), and efficacy (97.6%) by multi-ROC analysis with AUC = 0.930.

It is noteworthy that the platelet count is a convenient marker of liver fibrosis in several hepatic diseases, such as nonalcoholic fatty liver disease, hepatitis B and C; nevertheless, there are studies reporting conflicting results as thrombocytopenia is valuable marker of advanced liver disease and combined assessment of the AST/ALT ratio and platelet had a high diagnostic value for cirrhosis [16].

## Conclusion

Serum GDF-15 levels can significantly differentiate between chronic HCV patients with liver fibrosis and healthy controls, and also can discriminate between early and advanced liver fibrosis. Therefore, GDF-15 can be used as an early predictor of liver fibrosis assisting physicians initiating treatment earlier hence achieving higher survival rate.

## Data Availability

All the data and material are available.

## Abbreviations

AFP: Alpha fetoprotein
ALT: Alanine transaminase
APRI: AST-platelet ratio index
AST: Aspartate transaminase
AUC: Area under curve
BMI: Body mass index
ECM: Extracellular matrix
ELISA: Enzyme-linked immunosorbent assay
FIB-4 index: Fibrosis-4 index
GDF-15: Growth differentiation factor 15
GGT: Gamma glutamyl transferase
HCV: Hepatitis C virus
HRP: Streptavidin-horse radish peroxidase
INR: International normalized ratio
MARSI: Medicine Ain Shams Research Institute
NAFLD: Nonalcoholic fatty liver disease
NPV: Negative predictive value
PPV: Positive predictive value
ROC: Receiver operating characteristic
TGF-β: Transforming growth factor beta
